# Disclosure of HIV Status and Social Support Among People Living With HIV

**DOI:** 10.5812/ircmj.11856

**Published:** 2014-08-05

**Authors:** Zahra Jorjoran Shushtari, Homeira Sajjadi, Ameneh Setareh Forouzan, Yahya Salimi, Masoumeh Dejman

**Affiliations:** 1 Health Research Center, University of Social Welfare and Rehabilitation Sciences, Tehran, IR Iran; 2 Social Determinants of Health Research Center, Department of Social Welfare Management, University of Social Welfare and Rehabilitation Sciences, Tehran, IR Iran; 3 Department of Epidemiology and Biostatistics, Public Health School, Kermanshah University of Medical Sciences, Kermanshah, IR Iran; 4 Department of Epidemiology and Biostatistics, Public Health School, Tehran University of Medical Sciences, Tehran, IR Iran

**Keywords:** Disclosure, HIV, Social Support, Iran

## Abstract

**Background:**

Disclosure of HIV is important for improving self-care behaviors, psychological well-being, commitment to the treatment, and reducing risk of transmission. One of the major benefits of disclosure is social support, which is an essential resource for effective coping with HIV infection. However, receiving any social support requires disclosing of HIV status.

**Objectives:**

This study aimed to determine the disclosure of HIV status and its related factors such as social support in addition to demographic and disease characteristics among people living with HIV in Iran.

**Patients and Methods:**

This cross-sectional study, using simple random sampling, was carried out on 175 people with HIV/AIDS who referred to Behavioral Counseling Centers. The self-administrated, Norbeck Social Support Questionnaire was used to measure social support. Disclosure of HIV status was assessed with an investigator-designed questions. Multiple logistic regression analysis with backward Likelihood Ratio method was applied to identify the adjusted odds ratio between disclosure as dependent variable and demographic variables, social support as independent variables.

**Results:**

Participants were often disclosed their HIV status to family members. But there were differences about disclosure of HIV status within the context of the family. Family members were perceived as more supportive. Multiple logistic regression analysis demonstrates that the gender (adjusted OR = 0.181; 95% CI .068-0.479), CD4 cell count (adjusted OR = 0.997; 95% CI 0.994-0.999), route of transmission (injection-drug user [adjusted OR = 9.366; 95% CI 3.358-26.123] and other routes [tattooing, mother to child, dental services, etc.], [adjusted OR = 3.752; 95% CI 1.157-12.167]), and functional support variable (adjusted OR = 1.007; 95% CI 1.001-1.013) remained in the model as significant predictors for disclosure.

**Conclusions:**

The results of this study regarding disclosure of HIV status and its relations to social support and some demographic variables can provide an understanding based on the evidence for promotion of knowledge and coping interventions about people living with HIV/AIDS and their perceived social support status.

## 1. Background

Disclosure of HIV status is a kind of decision. Each person with HIV may decide that with whom he/she wants to disclose his/her status for requesting help, information and support. Based on the literature, disclosure of HIV is important for improving self-care behaviors, psychological well-being (1), adherence to treatment (2), and reducing risk of transmission of infection (3, 4). However, previous research has shown that, due to negative attitude of society and HIV-related stigma, disclosure of HIV-status is a critical concern among People Living With HIV (PLWH) especially in developing countries (1). Many of them are often reluctant to disclose because of fear of negative reactions such as rejection, exclusion, discrimination, and even assault that ultimately result in loss of social support from their social network (1, 5, 6).

Disclosure of HIV-status is typically a selective process that occurs based on cognitive appraisal of honesty and confidence from others and perceived determinants and benefits to PLWH (1, 7). One of the major benefits of disclosure can be social support which is an essential resource for coping effectively with HIV infection (1, 8, 9), although it has not been documented in some studies (10). Social support is an important aspect of psychological adjustment that can promote well-being for many people living with HIV, but receiving social support is required to disclose HIV status from PLWH (9). Researchers have reported that disclosure of HIV status varies with regard to the types of relationships (7, 11, 12). However, there is no consistency among their results. Some of the studies suggest that PLWH intend to disclose HIV-status to family members more than friends and coworkers. Whereas, some other studies showed that the disclosure was more towards the friends (7, 13). Contrary to other countries, particularly western society, information about social support and disclosure of HIV status is limited in Iran. Also, based on the literature, the statistical models have been used a little for assessing adjusted associations between disclosure of HIV-status and related factors (9, 14).

## 2. Objectives

This study aimed to determine the disclosure of HIV status and its related factors as social support among PLWH in Iran.

## 3. Patients and Methods

### 3.1. Study Site and Procedure 

In the present cross-sectional study, 175 participants , selected using simple random sampling, were selected from PLWHs, who referred to Behavioral Counseling Centers (BCCs) of Tehran University of Medical Sciences from June to December of 2011. BCCs of Tehran University of Medical Sciences are among major centers that provide free counseling, prevention, and treatment services for people with high-risk behaviors, HIV-positive and patient with AIDS. Many of the people with HIV/AIDS are covered by these centers. For eligibility, respondents had to be over 15 years old, being literate, HIV-positive, and being aware of HIV status at least six months earlier. The last criterion was chosen because the duration of time on the point of diagnosis may have an impact on the number or type of people to whom the PLWH decides to “disclose” (15). Informed consent from all participants was obtained. The present study was reviewed and approved by Research Ethics Board at the University of Social Welfare and Rehabilitation of Tehran.

### 3.2. Measures

The self-administrated Norbeck Social Support Questionnaire (NSSQ) was used to measure social support. The NSSQ includes five subscales: emotional support, aid support, functional support (sum of emotional and aid support; range = 0 − 384), structural support (sum of network size, duration and frequency of contact; range = 0 − 240), and loss support (0 − 120). The fifth subscale" total loss", was eliminated because participants had varied responses with “too missing”, which could not be reported. The reliability and validity of the NSSQ were confirmed in other studies (16, 17). Reliability and validity of the Farsi version of the NSSQ have been evaluated and approved (18). In the present study, Cronbach α scores for subscales have ranged from 0.86 to 0.95.

To assess HIV status disclosure, participants were asked with an investigator-designed questions. At first, they were asked, whether they disclosed their HIV-status to anybody. If the answer is yes, they were asked to indicate their relationships such as, mother, father, spouse and so on. Another question was, whether the participants perceived any social support from them (See the section of social support measure).

Also, participants were asked about socio-demographic characteristics, including age, gender, marital status, level of education, number of household members, CD4 cell counts and route of transmission of infection. Data for CD4 cell counts were gathered from medical records.

### 3.3. Data Analysis

The descriptive analyses were used to assess disclosure of HIV-status for each relationship type. After the normality assumption was proven, t test was used for comparison the mean of social support types between PLWH with disclosed and non-disclosed ones. Also multiple logistic regression with backward Likelihood Ratio method was applied to identify the adjusted association between demographic variables, social support as independent variables ,where the significant level of their association with disclosure was lower than 0.2 in the bivariate analysis, and disclosure as the dependent variable. The following variables were entered into the model as the dummy variables: route of transmission of HIV, socio-economic status and marital status. The baseline level for these variables was 'sexual relationship', 'low', and 'married', respectively. The Hosmer-Lemeshow test was applied in order to assess goodness of fit of the final model. Data were analyzed by using Statistical Package for the Social Sciences (SPSS) version 11.5. Significant level was at 0.05.

## 4. Results

Response rate of the participants was nearly 95%. The mean age of the participant in this sample (N = 175) were 36(SD = 8.4) year. The majority of them were male 65.1% and married 48.6%. Most participants reported sexual relationship as the route of transmission (45.8%). Demographic characteristics of participants were presented in detail (Table 1).

**Table 1.** Demographic and disease characteristics of Participants (n = 175)

Variable	No.	Mean ± SD or Percentage
**Age**	175	36 **± **8.4
Range (min - max)		20 - 67
**Number of years of education**	175	9.3 **± **3.95
Range (min - max)		2 - 23
**CD4 cell count**	175	314 **± **258
Range (min - max)		9 - 2104
**Route of transmission**		
Sexual relationship	80	45.8%
Injection-drug user	69	39.4%
Other (tattooing, mother to child, dental services)	26	14.8%
**Marital status**		
Married	85	48.6%
Single	47	26.8%
Divorced	43	24.6%
**Gender**		
Female	61	34.9%
Male	114	65.1%
**Socio-economic status**		
Low	12	6.9%
Moderate	118	67.4 %
High	45	25.7%

Table 2 shows the disclosure pattern among various relationships. Among the participants, 73.1% (N = 128) had disclosed their HIV status to at least one person of their social network. For example; 21.1% (N = 37) of participants disclosed their HIV status to their fathers. 2.8% of them had not disclosed their HIV status to any family members, and 42.9% had not disclosed to any friends. Furthermore, 26.9% (N = 47) of participants had not disclosed their HIV status to any network members. Participants disclosed their HIV status to family members more than to friends. As shown in the Table 3, the mean of various types of perceived social support among all participants, who disclosed or not disclosed their HIV−status, considering the attainable range of these subscales were low, but the mean of social support among participants with disclosure was greater than participants who had not disclosed to, although these differences were not significant. Family members who were aware of the participant’s HIV−status were significantly perceived as more emotional and supportive than others who were not disclosed to (P = 0.03). The multiple logistic regression analysis demonstrates the adjusted associations of independent variables with the disclosure of HIV−status as the dependent variable. As shown in Table 4, the gender (P = 0.001), CD4 count cell (P = 0.035), route of transmission, including of injection-drug user and other (such as tattooing, mother to child, dental services, etc.) (P = 0.000) and functional support variable (P = 0.031) remained in the model as significant predictors for disclosure of HIV−status. Hosmer−Lemeshow test showed that goodness of fit of model was good (P = 0.482).

**Table 2.** Disclosure Pattern Among Network Members of Participants (n = 175)

Variable	Value ^a^
**Non-disclosed**	47 (26.9)
**Disclosed**	128 (73.1)
**Family**	
Father	37 (21.1)
Mother	71 (40.5)
Brother	63 (36)
Sister	77 (44)
Spouse ^b^	76 (59.3)
**Friend**	6 (2.8)
**Family and friend**	45 (25.7)
**All network members**	5 (2.8)

^a^ Data are presented as No. (%).

^b^ Without single and divorced people.

**Table 3.** Social Support Among Network Tie of Participants (n = 175) ^a^

	Disclosed	Non-Disclosed	P Value
**Family**			
Emotional support ^b^	77.67 **± **50.94	61.97 **± **33.14	0.030
Aid support ^c^	34.73 **± **22.63	30.81 **± **19.59	0.509
Functional support ^d^	112.4 **± **69.03	92.78 **± **51.67	0.204
Structural support ^e^	63.12 **± **33.81	55.21 **± **24.88	0.185
**Friend**			
Emotional support	18.99 **± **13.85	19.55 **± **24.02	0.916
Aid support	9.15 **± **7.60	8.48 **± **7.7	0.788
Functional support	28.14 **± **18.96	28.03 **± **26.47	0.973
Structural support	17.40 **± **8.81	18.21 **± **16.11	0.964
**Total network members**			
Emotional support	89.70 **± **53.86	81.52 **± **42.09	0.280
Aid support	44.09 **± **27.73	39.29 **± **20.91	0.281
Functional support	133.79 **± **71.76	120.81 **± **63.28	0.061
Structural support	81.09 **± **40.33	73.42 **± **27.43	0.091

^a^ data are presented as Mean **± **SD.

^b^ Attainable range = 0−576.

^c^ Attainable range = 0−384.

^d^ Attainable range = 0−192.

^e^ Attainable range = 0−240.

**Table 4.** Logistic Regression Analysis for Prediction of Disclosure in Participants (n = 175)

	Unadjusted OR (95% CI)	P Value	Adjusted OR (95% CI)	P Value
**Gender**				
Female	1		1	
Male	0.553 (0.262 - 1.166)	0.119	0.181 (0.068 - 0.479)	0.001
**CD4 cell count**	0.996 (0.993 - 0.999)	0.015	0.997 (0.994 - 0.999)	0.035
**Route of transmission**				
Sexual relationship	1		1	
Injection-drug user	2.7 (1.25 - 5.84)	0.012	9.366 (3.358 - 26.123)	0.000
Other (tattooing, mother to child, dental services)	1.9 (0.684 - 5.25)	0.219	3.752 (1.157 - 12.167)	0.028
**Functional support**	1.05 (1 - 1.01)	0.061	1.007 (1.001 - 1.013)	0.031
**Socio-economic status**				
Low	1		1	
Moderate	0.191 (0.024 - 1.53)	0.120	0.246 (0.029 - 2.087)	0.199
High	0.42 (0.047 - 3.73)	0.437	0.727 (0.075 - 7.016)	0.783

## 5. Discussion

This study, based on our literature review is one of the few studies that determines the pattern of HIV−status disclosure and perceived social support among people with HIV in Iran. Results of this study provide an understanding of what factors are associated with disclosure practices of PLWH to members of their social networks. The findings showed that more than half of the participants disclosed their HIV status at least to one of their personal network members that is consistent with the result of other studies in the USA and Africa (15). HIV status was often disclosed to the family members significantly more than friends. Based on the results, there were differences about disclosure of HIV status within the context of the family. Sister and mother were more disclosed among family members by participants, respectively. However, consistent with other studies, fathers were less disclosed among family members (7). It means that participants trust their mothers and sisters more than others. Therefore, it makes sense that, participants share their HIV status more with their family members who were considerably perceived as more supportive.

It can be concluded that disclosure pattern may be different among PLWH based on the barriers and needs associated with specific types of relationships. Furthermore, this finding can be the result of cultural values of the society that may play an important role in disclosure pattern. Iranian society is characterized by unique environmental, cultural, and political settings that are reflected in the nature of their social relations. Therefore, the context of study is an important factor that must be considered for interpreting the results. Also, our findings suggest that PLWH who have disclosed their HIV status to their network members perceived more social support from those relationships than they have not been disclosed to (this finding is consistent with literature) (9).

As the findings of logistic regression showed, the perceived social support, gender, CD4 cell counts and route of transmission of infection had significantly associated with the disclosure of HIV-status after adjustment for other variables in the model. This finding was consistent with the result of past studies (19, 20). Despite the significant adjusted association between social support and disclosure variable, the odds ratio of this association was not strong. Thus, with increase in social support scores, the odds of disclosure of HIV-status (although small) will increase too. This finding is consistent with the evidence (9); however, it is not stable among studies (14, 19). A reason for this inconsistent finding may be due to different measurements of social support. Logistic regression model revealed that participants who had lower CD4 cell counts were more likely to disclose, which is consistent with the previous research (21, 22). It may be for fear of isolation and loss of people to care for and support in advanced stages of the disease that increases the probability of disclosure among participants who had lower CD4 cell counts. Several studies have pointed to the role of gender in the disclosure. In this study, gender was a determinant of disclosure of HIV-status (23, 24). Thus, men have disclosed their HIV-status almost 80% less than women.

It must be mentioned that the results of the present study must be interpreted with regard to some methodological limitations. Our results relied entirely on self-report to assess disclosure of HIV-status to family, friend and other social network members and perceived social support from them. This study also used a cross-sectional design; therefore, it is impossible to draw causal inferences. Because this study targeted PLWHA (People Living With HIV/AIDS), referring to BCC of Tehran University of Medical Sciences with the mentioned condition, the findings cannot be generalized to other PLWHA. In addition, our sample was one of the convenience type and cannot be considered representative of people living with HIV.

A disclosure decision is based on identifying safe situations for disclosure. It is associated with how social network members perceive their new identities and their conditions in their networks. All people, including PLWH and their social network members may benefit from interventions that assist this continuous process, because the disclosure decision can be an important factor for social support. The family members may benefit from supportive interventions from persons who live with HIV patients. This support itself can improve the psychological and social conditions of PLWH. Moreover, social support of PLWH can also help to increase the advantageous disclosures of HIV-status and decrease its negative experiences. Therefore, interventions are needed to assist PLWH and their network members to manage the new conditions and increase the potential positive outcomes that can occur with the disclosure of HIV-status.
